# The effects of wearing facemasks on oxygenation and ventilation at rest and during physical activity

**DOI:** 10.1371/journal.pone.0247414

**Published:** 2021-02-24

**Authors:** Steven L. Shein, Sofie Whitticar, Kira K. Mascho, Elizabeth Pace, Richard Speicher, Kathleen Deakins

**Affiliations:** 1 Division of Pediatric Critical Care Medicine, Rainbow Babies and Children’s Hospital, Cleveland, Ohio, United States of America; 2 Department of Pediatrics, Rainbow Babies and Children’s Hospital, Cleveland, Ohio, United States of America; 3 Department of Respiratory Care, Rainbow Babies and Children’s Hospital, Cleveland, Ohio, United States of America; Vanderbilt University Medical Center, UNITED STATES

## Abstract

**Background:**

Facemasks are recommended to reduce the spread of SARS-CoV-2, but concern about inadequate gas exchange is an often cited reason for non-compliance.

**Research question:**

Among adult volunteers, do either cloth masks or surgical masks impair oxygenation or ventilation either at rest or during physical activity?

**Study design and methods:**

With IRB approval and informed consent, we measured heart rate (HR), transcutaneous carbon dioxide (CO_2_) tension and oxygen levels (SpO_2_) at the conclusion of six 10-minute phases: sitting quietly and walking briskly without a mask, sitting quietly and walking briskly while wearing a cloth mask, and sitting quietly and walking briskly while wearing a surgical mask. Brisk walking required at least a 10bpm increase in heart rate. Occurrences of hypoxemia (decrease in SpO_2_ of ≥3% from baseline to a value of ≤94%) and hypercarbia (increase in CO_2_ tension of ≥5 mmHg from baseline to a value of ≥46 mmHg) in individual subjects were collected. Wilcoxon signed-rank was used for pairwise comparisons among values for the whole cohort (e.g. walking without a mask versus walking with a cloth mask).

**Results:**

Among 50 adult volunteers (median age 33 years; 32% with a co-morbidity), there were no episodes of hypoxemia or hypercarbia (0%; 95% confidence interval 0–1.9%). In paired comparisons, there were no statistically significant differences in either CO_2_ or SpO_2_ between baseline measurements without a mask and those while wearing either kind of mask mask, both at rest and after walking briskly for ten minutes.

**Interpretation:**

The risk of pathologic gas exchange impairment with cloth masks and surgical masks is near-zero in the general adult population.

## Introduction

More than 2 million people have died during the Coronavirus disease 2019 (COVID-19) pandemic [1]. The limited therapeutic options with possible efficacy include corticosteroids [2], antivirals [3], and monoclonal antibodies [4]. Widespread vaccination is not expected for several more months, at least [5]. Until then, more rudimentary non-pharmacologic measures may be the most effective way to reduce the global death toll, including social distancing, hand washing, and wearing facemasks.

Facemasks are recommended to reduce the chances that the wearer spreads SARS-CoV-2 [6], and may provide some protection for the person wearing the mask as well [7, 8]. But, widespread usage of facemasks is limited largely by feeling they are uncomfortable and concerns of inadequate gas exchange [9]. In small studies (n≤20), medical facemasks did not cause clinically significant changes in heart rate, oxygenation, or ventilation [10, 11]. Cloth facemasks now commonly being used have not been thoroughly studied. Among 25 elderly volunteers, cloth masks did not cause significant hypoxemia during usual daily activities in one recent study [12]. In our larger study of a more generalizable cohort, we tested the effects of cloth and surgical facemasks on heart rate, oxygen saturation and transcutaneous carbon dioxide levels both at rest and during physical activity.

## Materials and methods

With approval from the University Hospitals of Cleveland IRB (study #20200804), we enrolled hospital employees in this study between August and October 2020. Participants were recruited by sending IRB-approved emails to multiple hospital distribution lists (e.g. Pediatric ICU nursing staff, Pediatric residents, etc.) with efforts to also include groups with different demographics (e.g. Surgery residents, secretarial staff, etc.) to make the cohort more representative. Written informed consent was obtained from each subject. Inclusion criteria were age 18–65 years old and ability to walk briskly for 10 minutes. Subjects unable to wear a facemask due to an underlying medical condition were not eligible. After provision of informed consent, a transcutaneous sensor (V-Sign Sensor 2; SenTec AG, Therwil, Switzerland) was applied to the subject’s skin per manufacturer’s recommendations. This sensor measures heart rate (HR; normal range: 60–100 beats per minute [bpm]), carbon dioxide tension (CO_2_; normal range: 35–45 mmHg) and oxygen saturation (SpO_2_; normal range: 95–100%). Measurements of each of the three parameters were allowed to stabilize over several minutes and then the study began.

The study consisted of six 10-minute phases, with measurements of all three parameters occurring at the conclusion of each phase. First, the subject sat quietly without wearing a mask. Second, the subject walked briskly without wearing a mask. Third, the subject sat quietly while wearing a cloth mask. Fourth, the subject walked briskly wearing a cloth mask. Fifth, the subject sat quietly while wearing a surgical mask. Finally, the subject walked briskly while wearing a surgical mask. Masks always covered the subject’s nose and mouth. Subjects brought their own cloth masks; surgical masks were those used at our hospital. HR, SpO_2_, and transcutaneous CO_2_ tension were each measured three times at 1-minute intervals as each phase concluded. During the brisk walking phase, subjects were encouraged to walk faster if their heart rate did not increase by at least 10 bpm from their baseline measurement.

The average of the three measurements of each variable was used for analysis. The primary outcome was the number of subjects with hypoxemia or hypercarbia associated with wearing a mask. Hypoxemia was defined as a decrease in SpO_2_of ≥3% from baseline to a value of ≤94% while wearing a mask. Hypercarbia was defined as an increase in CO_2_ tension of ≥5 mmHg from baseline to a value while wearing a mask of ≥46 mmHg. These definitions were chosen to ensure that a patient had both a substantial change from their baseline and a measurement that is outside the normal range. Enrolling 50 subjects would enable estimating these rates with a 95% confidence interval of approximately ±2%. In addition, Wilcoxon signed-rank tests were used to compare paired values (e.g. maskless while walking versus cloth mask while walking) for the group as a whole using SigmaPlot v12.5 (Systat Software, San Jose, California). Data are reported as n (%) or median (interquartile range), and p < 0.05 (with Bonferroni correction for four comparisons) defined statistical significance.

## Results and discussion

We enrolled 50 subjects, 34 (68%) of whom are female, with a median age of 33 (29–44.75) years (Table 1). Sixteen subjects reported they had a significant co-morbidity, most commonly asthma (n = 6) and hypertension (n = 4). While sitting (Table 2), baseline heart rate while not wearing a mask was 73.2 (67.0–79.4) bpm, baseline SpO_2_ was 97.3% (96.6–98.1%) and baseline CO_2_ tension was 38.8 (35.7–44.1) mmHg. While walking, baseline values were 101.2 (89.0–111.8) bpm, 97.2% (96.3–98.0%), and 39.8 (37.0–43.7) mmHg, respectively. The increase in heart rate with walking was statistically significant compared to sitting, but the differences in SpO_2_ and CO_2_ tension were not. No subjects were unable to complete testing due to dyspnea or any other reason.

**Table 1:** Demographics.

Variable	n (%) or median (IQR)
Female	34 (68%)
Age	33 (29–44.75) years
Co-morbid conditions*	16 (32%) (any)
- Asthma	6 (12%)
- Hypertension	4 (8%)
- Hypothyroidism	3 (6%)
- Hypercholesterolemia	2 (4%)
- Other	1 each of arrhythmia, acid reflux, mixed connective tissue disorder

* some patients had >1 co-morbid condition.

**Table 2:** Vital signs at baseline (no mask).

Variable	While sitting	Walking briskly	p-value
Heart rate (bpm)	73.2 (67.0–79.4)	101.2 (89.0–111.8)	<0.001
SpO_2_	97.3% (96.6–98.1%)	97.2% (96.3–98.0%)	0.235
CO_2_ tension (mmHg)	38.8 (35.7–44.1)	39.8 (37.0–43.7)	0.156

For each of the three vital signs assessed, there were a total of 200 paired comparisons among the 50 subjects. Zero subjects (0%; 95% confidence interval: 0–1.9%) developed either hypoxemia or hypercarbia while wearing either type of mask, either at rest or while walking. One subject had SpO_2_ decrease by ≥3% (99% to 95.7% while walking with a cloth mask). Five subjects had CO_2_ tension increase by ≥5 mmHg, one of whom had this occur during three phases (walking with a cloth mask, both sitting and walking with a surgical mask). The average CO_2_ tension during the seven instances that it increased by ≥5 mmHg from baseline was 42.2 mmHg.

**Fig 1:**
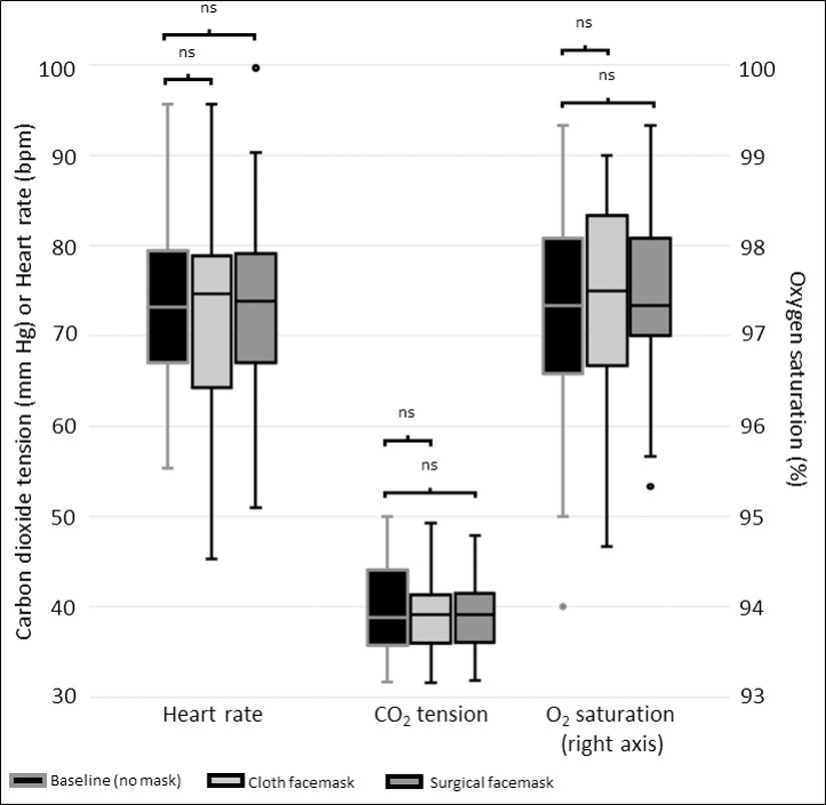
Measurements taken at rest. Boxes represent the 25^th^-75^th^ percentile of each variable. The horizontal line in each box represents the median. The whiskers represent the local maximum and local minimum values. Values that are >1.5 times the interquartile range from either end of the box are considered outliers and denoted with a small circle. Paired comparisons were performed using the Wilcoxon signed rank test. At rest, all comparisons between baseline/maskless values (black boxes) and facemask values (gray boxes) were not statistically significant (“ns”).

Compared to sitting without a mask, there were no statistically significant differences observed while sitting wearing a cloth mask in heart rate (74.7 [64.3–78.8] vs. 73.2 [67.0–79.4] bpm, p = 0.829), in SpO_2_ (97.3% [96.6–98.1%] vs. 97.5% [96.7–98.3%], p = 0.769), or in CO_2_ tension (39.1 [35.9–41.3] vs. 38.8 [35.7–44.1] mmHg, p = 0.209) (Fig 1). Values obtained when sitting while wearing a surgical mask (73.8 [67.0–79.1] bpm, 97.3% [97.0–98.1%], and 39.1 [36.1–41.5] mmHg, respectively) also did not significantly differ from baseline values (all p-values >0.178).

**Fig 2:**
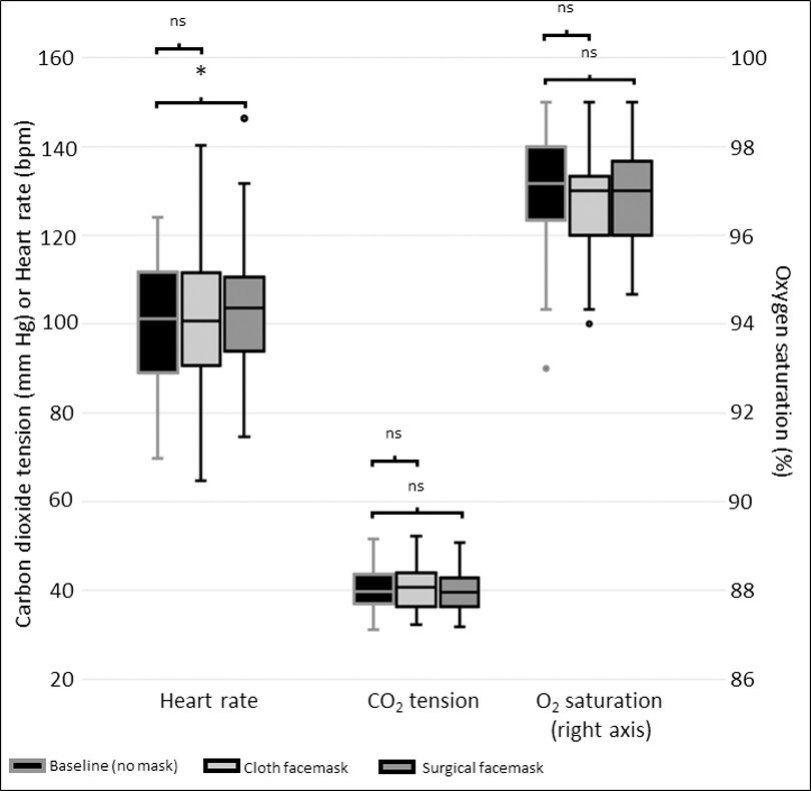
Measurements taken during physical activity. Boxes represent the 25^th^-75^th^ percentile of each variable. The horizontal line in each box represents the median. The whiskers represent the local maximum and local minimum values. Values that are >1.5 times the interquartile range from either end of the box are considered outliers and denoted with a small circle. Paired comparisons were performed using the Wilcoxon signed rank test. Non-significant differences are denoted “ns” and statistically significant differences are denoted with an asterisk.

Compared to walking without a mask, there were no statistically significant differences observed while walking wearing a cloth mask in heart rate (100.7 [90.7–111.6] vs. 101.2 [89.0–111.8] bpm, p > 0.999), in SpO_2_ (97.0% [96.0–97.3%] vs. 97.2% [96.3–98.0%], p = 0.165), or in CO_2_ tension (40.7 [36.3–43.9] vs. 39.8 [37.0–43.7], p = 0.999) (Fig 2). Walking with a surgical mask did lead to a modest increase in heart rate (103.7 [93.8–110.7] vs. 101.2 [89.0–111.8] bpm, p = 0.016), but no significant changes in SpO_2_ (97.0% [96.0–97.7%], p = 0.603) or CO_2_ tension (39.6 [36.3–42.8] mmHg, p = 0.618).

In total, there were 200 measurements of CO_2_ tension, all done in triplicate. The difference between the minimum of these three measurements and the maximum of the set was less that 2 mmHg in >95% of instances.

Among 50 hospital employees, 32% of whom had a co-morbidity, no subjects developed hypoxemia or hypercarbia while wearing a cloth mask or a surgical mask at rest or during activity. Neither cloth masks nor surgical masks significantly affected heart rate, oxygen saturation, or CO_2_ tension while at rest. During physical activity that was strenuous enough to increase heart rates by approximately 30 bpm, there were again no significant changes in gas exchange when wearing either type of facemask. Overall, these findings may help people–especially those unaccustomed to wearing facemasks–be reassured that their body is able to adequately get oxygen in and carbon dioxide out while wearing a facemask, and therefore may help increase usage and limit viral spread.

We observed two statistically significant changes in physiology during our study, both changes in heart rate and not gas exchange. First, we observed an expected and intended increase in heart rate with walking without a mask compared to sitting without a mask. This finding supports that the level of activity achieved by our subjects was considerable, though future studies may use treadmills or other devices to more precisely prescribe the level of exertion. Second, the median heart rate while walking with a surgical mask was 2.5 bpm higher than the median heart rate while walking without a mask, a difference that is likely clinically insignificant. No statistically significant changes in gas exchange were observed when values for the whole cohort were compared. On an individual subject level, no subjects met our definitions of hypoxemia or hypercarbia, and the few subjects with a notable change in either SpO_2_ or CO_2_ tension stayed within the normal range.

Prior studies of smaller cohorts have similarly found that surgical facemasks cause statistically insignificant [13] or clinically insignificant [14] effects on heart rate and gas exchange. The COVID-19 pandemic has led to increased interest in studying the effects of masks on gas exchange, including cloth masks. In one study of 25 elderly volunteers (average age 77±6 years; 36% with comorbidities), no episodes of hypoxemia (defined as SpO_2_ <92%) occurred during activities of daily living; hypercarbia was not assessed. Another study tested surgical masks in 15 healthy young adults (average age 31±2 years; 0% with comorbidities) and 15 veterans with severe chronic obstructive pulmonary disease (average age 72 years). Though formal statistical analyses were not reported, the authors observed “no major changes in [end-tidal] CO_2_ or SpO_2_ of clinical significance” while resting wearing a surgical mask in either patient group. However, mean SpO_2_ decreased by 2.3±7.3% in patients with pulmonary disease while performing a 6-minute walk test in a surgical mask, but no “major physiologic changes” in CO_2_ retention were reported. As in these two studies, we found that the risk of hypoxemia is exquisitely low, though our findings from a larger cohort with a wider age range and representative rate of comorbidities may be more generalizable. We also uniquely report that the risk of hypercarbia from cloth masks is minimal.

Beyond the larger sample size, advantages of our study include testing cloth facemasks that are actually being used by people in day-to-day life during the current pandemic, not excluding subjects with common co-morbidities like asthma [15], and measuring ventilation and not just oxygenation [12]. Our study has limitations that could be addressed in future work. First, our sample size is modest, though notably larger than many prior studies assessing gas exchange while wearing masks. Second, the duration of each study phase was 10 minutes, which was chosen to provide adequate time to observe physiologic changes but not require people to volunteer more than 90 minutes of their time. Though the substantial increase in heart rate with walking supports that the duration and intensity were sufficient, future studies may consider a longer duration and/or higher intensity of physical activity. Similarly, the rigor of the activity could be better controlled by using a treadmill. Third, the order of testing could be randomized to make sure that vitals obtained during the last phases (i.e. wearing the surgical mask) were not influenced by the subjects being tired from the prior phases. However, each subject had a 10 minute period of rest (sitting) before each walking phase during which their heart rate returned to baseline, so it is unlikely that the slight increase in heart rate observed with surgical masks was due to subject fatigue. Fourth, we used transcutaneous measurements of CO_2_ tension rather than arterial blood sampling in order to minimize pain for the subjects, which may be a less accurate method of measurement. However, the SenTec monitor is validated as a surrogate for arterial blood sampling [16] and the measurements taken in triplicate in our study subjects were very consistent (almost always within 1–2 mmHg of each other).

## Conclusion

In conclusion, facemasks did not impair oxygenation or ventilation among 50 adults at rest or during physical activity. No episodes of hypoxemia or hypercarbia occurred with either cloth or surgical masks, both at rest and while walking briskly. The risk of pathologic gas exchange impairment with cloth masks and surgical masks is near-zero in the general adult population.

## Abbreviations

Bpm: beats per minute
CO_2_: = carbon dioxide
COVID-19: coronavirus disease 2019
HR: heart rate
SpO_2_: oxygen saturation
